# A new species of *Longicoelotes* (Araneae, Agelenidae) from China, with the first description of the male of *L. kulianganus* (Chamberlin, 1924)

**DOI:** 10.3897/zookeys.686.11711

**Published:** 2017-07-25

**Authors:** Xiaoqing Zhang, Zhe Zhao

**Affiliations:** 1 Institute of Zoology, Chinese Academy of Sciences, Beijing 100101, China; 2 Southeast Asia Biodiversity Research Institute, Chinese Academy of Sciences, Yezin, Nay Pyi Taw 05282, Myanmar

**Keywords:** East Asia, description, Coelotinae, taxonomy

## Abstract

A new *Longicoeletes* species is described from Jiangxi Province, China: *L. geei* **sp. n.** (♂♀). In addition, the male of *L. kulianganus* (Chamberlin, 1924) is described for the first time. DNA barcodes of the two species are documented for future use and as proof of molecular differences between these species.

## Introduction

The *Longicoelotes* was described by Wang (2002), with *L. karschi* Wang, 2002 from China as the type species. Wang (2003) transferred *Coelotes kulianganus* Chamberlin, 1924 from China and *C. senkakuensis* Shimojana, 2000 from Ryukyu Islands to *Longicoelotes*. Three species of *Longicoelotes* were known before the current study (World Spider Catalog 2017), two of which are restricted to China. This paper provides the description of a new species based on newly collected material from Jiangxi Province, China. It also redescribes *L. kulianganus* based on specimens collected from the type locality.

## Material and methods

Specimens were examined and measured with a Leica M205C stereomicroscope. Images were captured with an Olympus C7070 wide zoom digital camera mounted on an Olympus SZX12 dissecting microscope. Epigynes and male palps were examined after dissection. Epigynes were cleared by boiling in 10% KOH solution before taking photos. All measurements are given in millimeters. Leg measurements are given as: total length (femur, patella + tibia, metatarsus, tarsus). Only structures (palp and legs) of the left side of the body are described and measured.

Terminology used for parts of copulatory organs in the text and figures follows Wang (2002) with some modifications. Abbreviations used in the text and figures are: ALE = anterior lateral eye; AME = anterior median eye; AME-ALE = distance between AME and ALE; AME-AME = distance between AME and AME; ALE-PLE = distance between ALE and PLE; d = dorsal; Fe = femur; Mt = metatarsus; p = prolateral; Pa = patella; PLE = posterior lateral eye; PME = posterior median eye; PME-PLE = distance between PME and PLE; PME-PME = distance between PME and PME; r = retrolateral; Ta = tarsus; Ti = tibia; v = ventral. References to figures in the cited papers are listed in lowercase (fig. or figs); figures from the present paper are noted with an initial capital (Fig. or Figs).

DNA barcodes were obtained for future use: a partial fragment of the mitochondrial gene cytochrome oxidase subunit I (COI) was amplified and sequenced for two species using Primers LCO1490-oono (5’-CWACAAAYCATARRGATATTGG-3’) (Folmer et al. 1994; Miller et al. 2010) and HCO2198-zz (5’-TAAACTTCCAGGTGACCAAAAAATCA-3’) (Folmer et al. 1994; Zhao and Li 2016). For additional information on extraction, amplification, and sequencing procedures, see Zhao et al. (2013). All sequences were blasted in GenBank; accession numbers are provided in Table 1.

**Table 1.** Voucher specimen infromation.

Species	GenBank accession number	Sequence length	Collection localities
*L. geei* sp. n.	MF347606	543bp	China: Jiangxi: Wuyuan
*L. kulianganus*	MF347607	630bp	China: Fujian: Fuzhou: Guling

All specimens (including molecular vouchers) are deposited in the Institute of Zoology, Chinese Academy of Sciences in Beijing (IZCAS).

## Taxonomy

### Genus Longicoelotes

Taxon classificationAnimaliaORDOFAMILIA

Wang, 2002

Longicoelotes Wang, 2002: 109. Type species Longicoelotes karschi Wang, 2002 from Jiangsu Province, China.

#### Composition.

Four species of *Longicoelotes* in total: they are *L. geei* sp. n. (♂♀), *L. karschi* (♂♀), *L. kulianganus* (♂♀) from China, and *L. senkakuensis* (Shimojana, 2000) (♀) from Ryukyu Islands.

#### Note.

*Longicolotes karschi* is very similar to *L. kulianganus* and could be its junior synonym.

### Longicoelotes geei

Taxon classificationAnimaliaORDOFAMILIA

http://zoobank.org/AD46BF47-3AB3-42AD-97D9-7E4E39D83D28

Figs 1 , 2 , 5 , 6

#### Type material.

**Holotype** ♂: China: Jiangxi Province: Wuyuan County, Lianhua Cave, N29°29'02", E117°36'53", 350 m, 4.XII.2016, X. Zhang; **Paratypes**: 3♀ 2♂, same data as holotype; 3♀, the same locality, N29°29'02", E117°36'53", 352 m, 20.V.2013, Y. Luo and J. Liu.

#### Etymology.

The species is named after Mr. Nathaniel Gist Gee. Mr. Gee was an American biologist who lived in China about 35 years. He contributed greatly to the development of biological research and education in China, including the establishment of the first biology department in the history of university education in China (Fu 2016). He collected the holotype of *L. kulianganus*.

#### Diagnosis.

**Figure 1.**
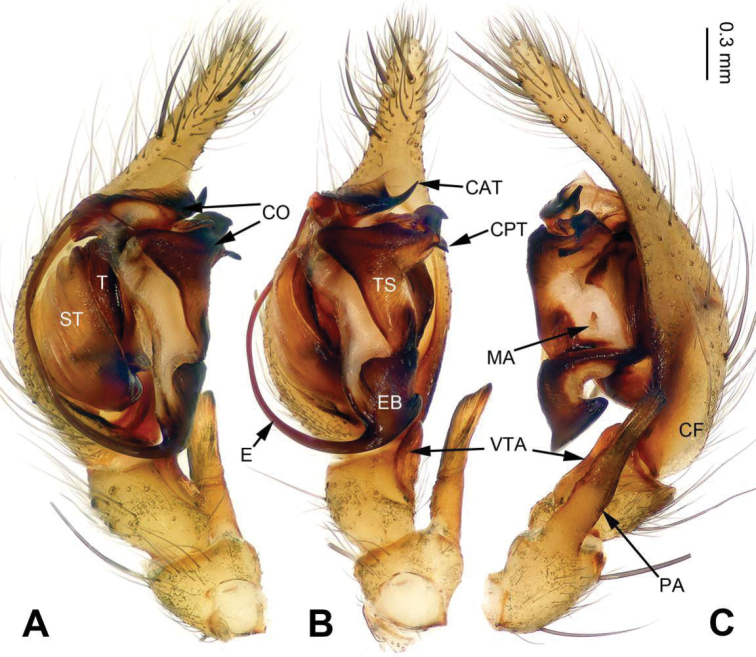
Palp of *Longicoelotes geei* sp. n., holotype male. **A** Prolateral view **B** Ventral view **C** Retrolateral view. CAT = anterior tip of conductor, CF = cymbial furrow, CO = conductor, CPT = posterior tip of conductor, E = embolus, EB = embolic base, MA = median apophysis, PA = patellar apophysis, ST = subtegulum, T = tegulum, TS = tegular sclerite, VTA = ventral tibial apophysis. Scale bar: equal for **A, B, C**.

**Figure 2.**
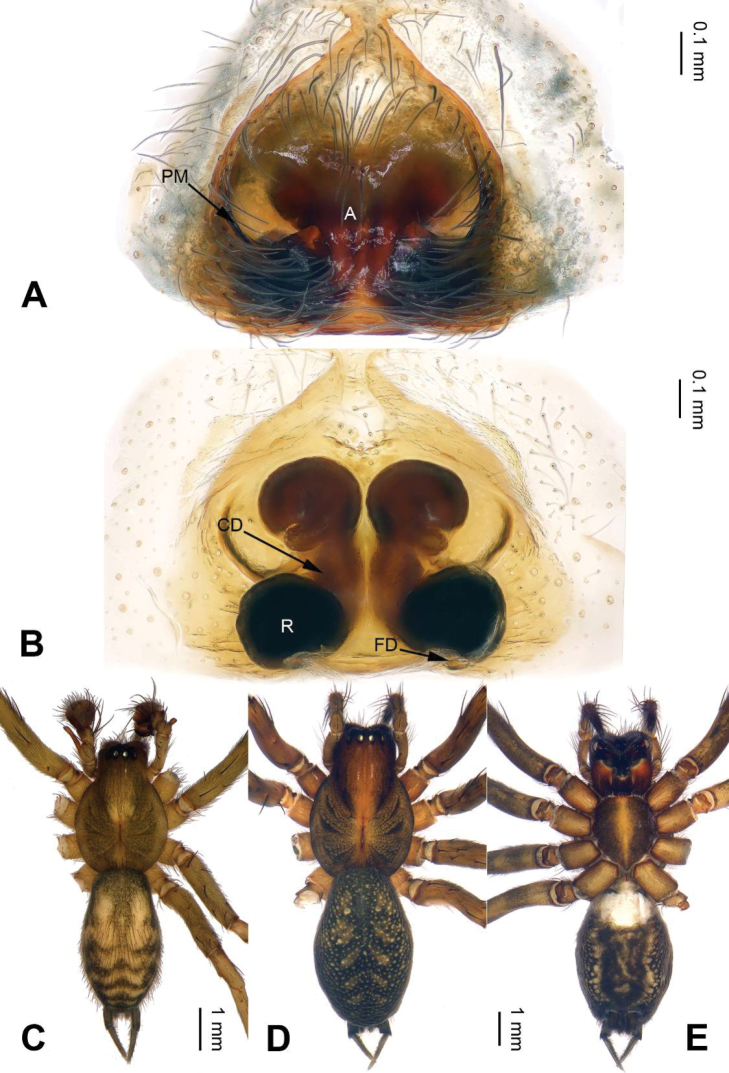
*Longicoelotes geei* sp. n., female paratype and male holotype. **A** Epigyne, ventral view **B** Vulva, dorsal view **C** Male habitus, dorsal view **D** Female habitus, dorsal view **E** Female habitus, ventral view. A = epigynal atrium, CD = copulatory duct, FD = fertilization duct, PM = posterior margin of atrium, R = receptacle. Scale bars: equal for **D, E**.

**Figure 3.**
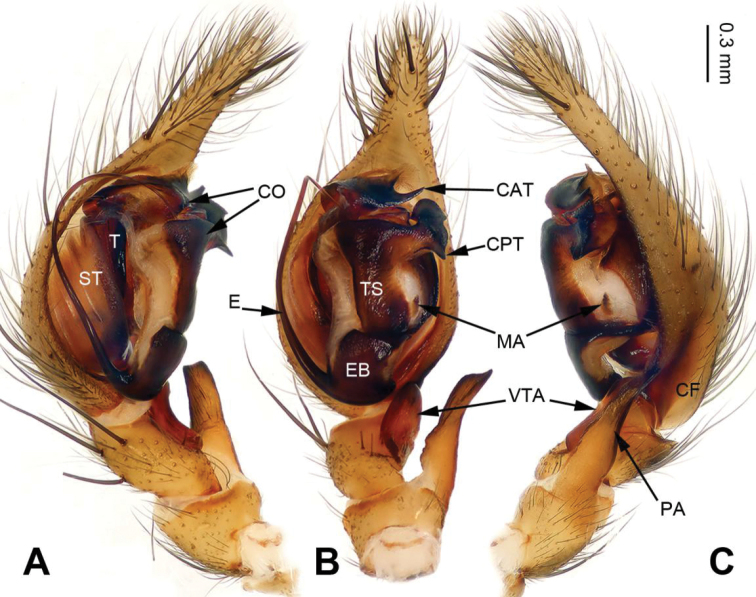
Palp of *Longicoelotes kulianganus*, specimen from Guling. **A** Prolateral view **B** Ventral view **C** Retrolateral view. CAT = anterior tip of conductor, CF = cymbial furrow, CO = conductor, CPT = posterior tip of conductor, E = embolus, EB = embolic base, MA = median apophysis, PA = patellar apophysis, ST = subtegulum, T = tegulum, TS = tegular sclerite, VTA = ventral tibial apophysis. Scale bar: equal for **A, B, C**.

**Figure 4.**
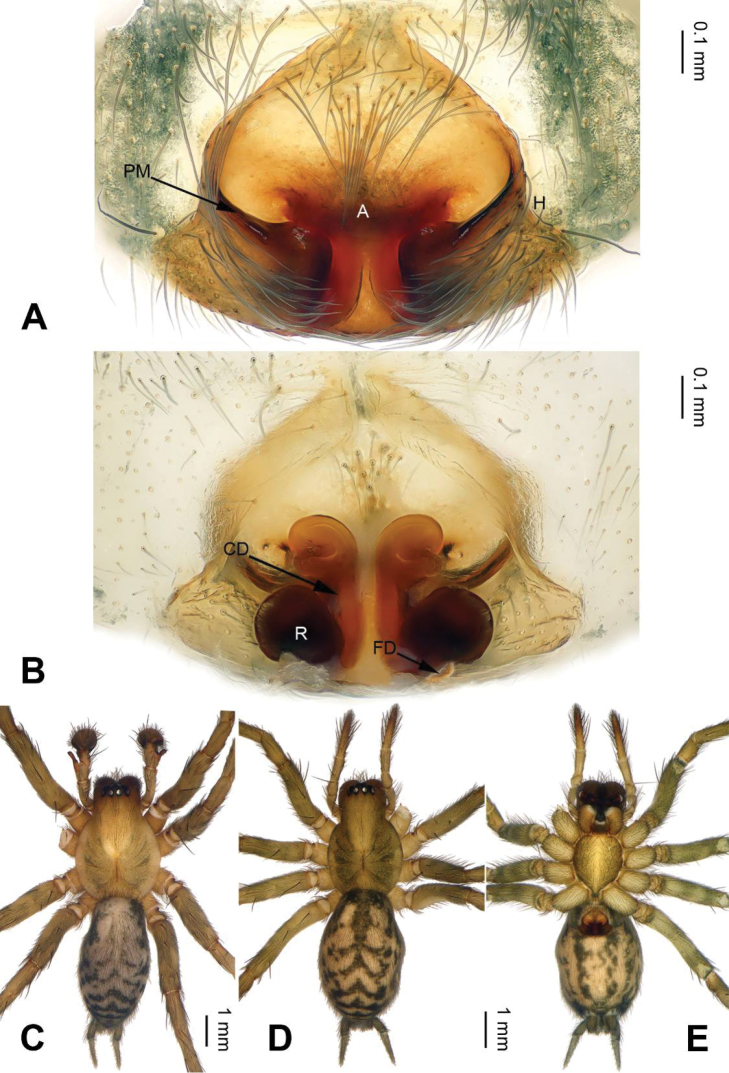
*Longicoelotes kulianganus*, specimen from Guling. **A** Epigyne, ventral view **B** Vulva, dorsal view **C** Male habitus, dorsal view **D** Female habitus, dorsal view **E** Female habitus, ventral view. A = epigynal atrium, CD = copulatory duct, FD = fertilization duct, H = epigynal hood, PM = posterior margin of atrium, R = receptacle. Scale bars: equal for **D, E**.

**Figure 5.**
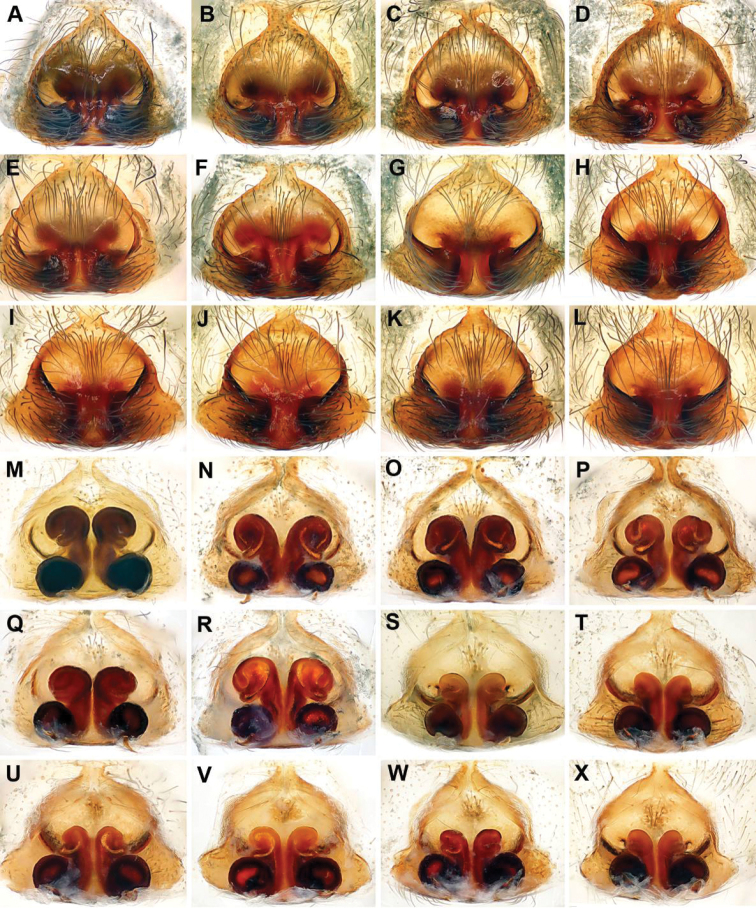
*Longicoelotes geei* sp. n. (**A–F, M–R)** and *L. kulianganus* (**G–L**, **S–X)**, variations in shape of epigynal atrium and size of copulatory ducts. **A–L** Epigyne, ventral view **M–X** Vulva, dorsal view.

The male can be distinguished from all other *Longicoelotes* species by its long cymbial furrow, about 1/3 length of cymbium (*vs* 1/4 or 1/5 in other species) (Figs 1, 3; Wang 2002: figs 312–314). The female differ from all other *Longicoelotes* species by the nearly heart-shaped atrium and large anterior parts of copulatory ducts, subequal to receptacles (*vs* nearly triangular atrium and small anterior parts in *L. kulianganus*, about 1/2 diameter of receptacles; nearly rounded atrium and extremely small anterior parts in *L. senkakuensis*, about 1/4 diameter of receptacles) (Figs 2, 4–5; Shimojana 2000: figs 46–49).

#### Description.

**Male (holotype)**: Total length 6.00. Carapace 2.50 long, 2.00 wide. Abdomen 3.50 long, 1.75 wide. Eye sizes and interdistances: AME 0.15, ALE 0.13, PME 0.15, PLE 0.10; AME-AME 0.05, AME-ALE 0.04, PME-PME 0.05, PME-PLE 0.06. Leg measurements: I: 12.45 (3.30, 4.00, 3.00, 2.15); II: 11.30 (3.25, 3.30, 2.75, 2.00); III: 10.65 (3.00, 3.25, 2.75, 1.65); IV: 14.65 (4.00, 4.25, 4.15, 2.25). Carapace greenish, with black lateral margins, the radial grooves distinct. Abdomen yellow, with blackish herringbone patterns. Palp as in Fig. 1: patellar apophysis long, about 2 times longer than tibia; tibia short, about 1/5 length of cymbium; cymbial furrow long, about 1/3 length of cymbium; ventral tibial apophysis as long as tibia, without pointed tip, extending beyond the tibia; conductor slender and bifurcated; posterior tip of conductor bifurcated; anterior tip of conductor bifurcated, ventral part sharp, dorsal part lamellate; median apophysis reduced; embolus beginning at 5:30 o’clock position.

Leg spination in male:

**Table:** 

	**Fe**	**Pa**	**Ti**	**Mt**	**Ta**
I	3d 2p 1r	-	1p 3-3v	2p 3-3v	-
II	3d 3p 2r	1d 1p	2p 3-3v	1p 3-3v	-
III	3d 2p 2r	2d 1p 1r	2d 2p 3r 3-3v	2d 5p 5r 3-3v	1p 1r
IV	3d 2p 1r	2d 1p 1r	2d 2p 2r 3-3v	5p 5r 3-3v	2p 1r

**Female (paratype)**: Total length 8.65. Carapace 4.10 long, 3.00 wide. Abdomen 4.55 long, 2.55 wide. Eye sizes and interdistances: AME 0.20, ALE 0.18, PME 0.18, PLE 0.15; AME-AME 0.08, AME-ALE 0.05, PME-PME 0.10, PME-PLE 0.10. Leg measurements: I: 14.25 (4.00, 5.00, 3.25, 2.00); II: 12.20 (3.50, 4.00, 3.00, 1.70); III: 11.35 (3.25, 3.75, 2.75, 1.60); IV: 15.95 (4.50, 5.10, 4.25, 2.10). Carapace reddish, with black lateral margins, the radial grooves distinct; sternum with light stripes. Abdomen black, with yellow spots and herringbone patterns. Epigyne as in Fig. 2: atrium nearly heart-shaped, length subequal to width; copulatory openings indistinct; copulatory ducts long, about 1.5 times longer than diameter of receptacles, touching each other; receptacles separated, about 1/2 diameter of receptacles; hoods indistinct.

Leg spination in female:

**Table:** 

	**Fe**	**Pa**	**Ti**	**Mt**	**Ta**
I	3d 2p 1r	-	1p 3-3v	1p 3-3v	-
II	3d 2p 2r	-	2p 3-3v	3p 1r 3-3v	-
III	3d 2p 2r	1d 1p	2d 2p 2r 3-3v	5p 5r 3-3v	1r
IV	3d 1p 1r	1d 1r	2d 2p 2r 3-3v	1d 5p 5r 3-3v	1p 1r

#### Distribution.

**Figure 6.**
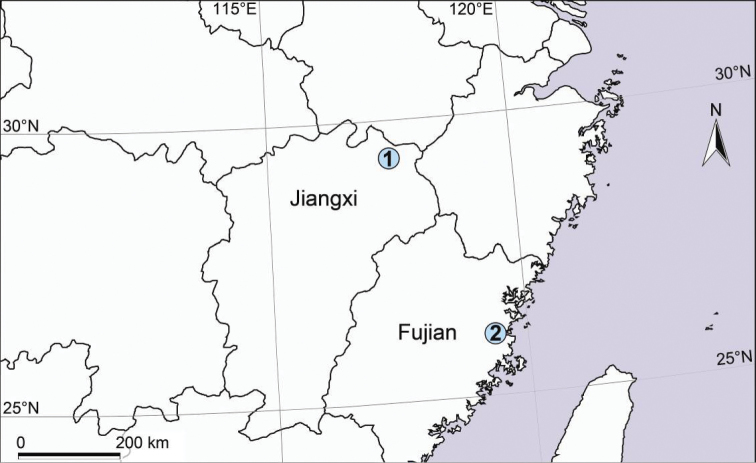
Collecting localities of two *Longicoelotes* species from China. **1**. *L. geei* sp. n., **2**. *L. kulianganus*.

Known only from Jiangxi Province of China (Fig. 6).

### Longicoelotes kulianganus

Taxon classificationAnimaliaORDOFAMILIA

(Chamberlin, 1924)

Figs 3 , 4 , 5 , 6

Coelotes kulianganus Chamberlin, 1924: 24, pl. 5, fig. 40 (♀).
Longicoelotes kulianganus : Wang 2003: 560 (transferred from Coelotes).

#### Material examined.

2♀ 3♂, China: Fujian Province: Fuzhou Prefecture: Guling (new name for Kuliang), Liushanwang Park, N26°05'34", E119°23'32", 725 m, 1.XII.2016, X. Zhang; 2♀ 8♂, the same locality, Yixia Villa, N26°05'32", E119°23'32", 718 m, 1.XII.2016, X. Zhang; 5♀ 3♂, the same locality, swimming pool, N26°05'33", E119°23'30", 684 m, 2.XII.2016, X. Zhang.

#### Diagnosis.

The male can be distinguished from other *Longicoelotes* species by indistinct median apophysis and short cymbial furrow, about 1/5 length of cymbium (*vs* 1/3 in *L. geei* sp. n.) (Figs 1, 3; Wang 2002: figs 312–314). The female can be separated from other congeners by its nearly triangular atrium and rounded anterior parts of copulatory ducts (*vs* nearly rounded atrium and extremely small anterior parts of copulatory ducts in *L. senkakuensis*) (Figs 4–5; Shimojana 2000: figs 46–49).

#### Description.

**Male**: Total length 8.00. Carapace 3.50 long, 2.75 wide. Abdomen 4.50 long, 2.50 wide. Eye sizes and interdistances: AME 0.20, ALE 0.18, PME 0.19, PLE 0.15; AME-AME 0.05, AME-ALE 0.05, PME-PME 0.08, PME-PLE 0.08. Leg measurements: I: 14.75 (4.25, 4.75, 3.50, 2.25); II: 12.35 (3.50, 4.00, 3.00, 1.85); III: 11.80 (3.30, 3.50, 3.25, 1.75); IV: 16.25 (4.50, 5.00, 4.50, 2.25). Carapace greenish, the radial grooves indistinct. Abdomen grayish, with blackish herringbone patterns. Palp as in Fig. 3: patellar apophysis long, about 2 times longer than tibia; tibia short, about 1/4 length of cymbium; cymbial furrow short, about 1/5 length of cymbium; ventral tibial apophysis subequal to the tibial length, without pointed tip; conductor broad and bifurcated; posterior tip of conductor bifurcated; anterior tip of conductor bifurcated, ventral part nearly spine-shaped, dorsal part lamellate; median apophysis indistinct; embolus beginning at 6:00 o’clock position.

Leg spination in male:

**Table:** 

	Fe	Pa	Ti	Mt	Ta
I	3d 2p 1r	-	3-3v	1p 3-3v	-
II	3d 1p 1r	-	2p 3-3v	3p 3-3v	-
III	3d 2p 2r	1d 1p 1r	2d 2p 2r 3-3v	5p 5r 3-3v	2p 1r
IV	3d 2p 1r	1d 1p 1r	2d 2p 2r 3-3v	5p 5r 3-3v	2p 1r

**Female**: Total length 6.75. Carapace 2.75 long, 2.15 wide. Abdomen 4.00 long, 2.25 wide. Eye sizes and interdistances: AME 0.15, ALE 0.15, PME 0.16, PLE 0.13; AME-AME 0.06, AME-ALE 0.05, PME-PME 0.10, PME-PLE 0.10. Leg measurements: I: 10.50 (3.00, 3.50, 2.50, 1.50); II: 9.20 (2.75, 3.00, 2.25, 1.20); III: 8.70 (2.50, 2.70, 2.25, 1.25); IV: 12.00 (3.50, 3.75, 3.25, 1.50). Carapace greenish, with black lateral margins, the radial grooves distinct; sternum with light stripes. Abdomen black, with yellow spots and herringbone patterns. Epigyne as in Fig. 4: atrium with well delimited posterior margin, length subequal to width; copulatory openings indistinct, hidden by posterior margin of atrium; copulatory ducts long, about 2 times longer than diameter of receptacles; receptacles widely separated, subequal to diameter receptacles; hoods distinct.

Leg spination in female:

**Table:** 

	Fe	Pa	Ti	Mt	Ta
I	3d 2p 1r	-	1p 3-3v	3-3v	-
II	3d 2p 2r	2d 1p	2p 3-3v	3p 1r 3-3v	-
III	3d 2p 2r	2d 1p 1r	2p 3r 3-3v	5p 5r 3-3v	1p 1r
IV	3d 1p 1r	1d 1p 1r	2d 2p 2r 3-3v	5p 5r 3-3v	1p 1r

#### Distribution.

Known only from Fujian Province of China (Fig. 6).

## Supplementary Material

XML Treatment for Longicoelotes

XML Treatment for Longicoelotes geei

XML Treatment for Longicoelotes kulianganus
